# A Ternary Inverter Based on Hybrid Conduction Mechanism of Band-to-Band Tunneling and Drift-Diffusion Process

**DOI:** 10.3390/mi15040522

**Published:** 2024-04-13

**Authors:** Bin Lu, Xin Ma, Dawei Wang, Guoqiang Chai, Yulei Chen, Zhu Li, Linpeng Dong

**Affiliations:** 1 School of Physics and Information Engineering, Shanxi Normal University, Taiyuan 030024, China; lubinsxnu@sina.cn (B.L.); maxin0043@163.com (X.M.);; 2 Shaanxi Province Key Laboratory of Thin Films Technology and Optical Test, Xi’an Technological University, Xi’an 710032, China

**Keywords:** ternary inverter, tunneling, drift-diffusion, hybrid conduction mechanism

## Abstract

In this paper, a novel transistor based on a hybrid conduction mechanism of band-to-band tunneling and drift-diffusion is proposed and investigated with the aid of TCAD tools. Besides the on and off states, the proposed device presents an additional intermediate state between the on and off states. Based on the tri-state behavior of the proposed TDFET (tunneling and drift-diffusion field-effect transistor), a ternary inverter is designed and its operation principle is studied in detail. It was found that this device achieves ternary logic with only two components, and its structure is simple. In addition, the influence of the supply voltage and the key device parameters are also investigated.

## 1. Introduction

In recent decades, driven by the pursuit of enhanced performance in binary logic chips and increased information density, the MOSFET (metal-oxide-semiconductor field-effect transistor) dimension has been rapidly reduced. However, the modulation of carrier thermionic emission over the energy barrier controlled by the gate bias poses challenges for MOSFETs in surpassing the sub-threshold swing (SS) limitation of 60 mV/dec at room temperature. This limitation leads to a rapid increase in the leakage current and power density during the miniaturization process [1,2,3,4]. Consequently, this presents a significant obstacle to further advancement in information density and circuit performance. Although steep slope devices, such as negative-capacitance FETs (NCFETs) [5,6,7,8,9] and tunneling FETs (TFETs) [10,11,12,13,14], can mitigate this issue, the number of bits is inherently smaller than the number of gates in a binary Boolean logic system [2].

Fundamentally, the most promising strategy for addressing the power challenge and significantly improving information density involves a shift from traditional binary logic to ternary logic [15,16,17,18,19]. In binary systems, information is represented by {0, 1}, whereas in a ternary system, it is represented by {0, 1, 2} or {−1, 0, 1}. Consequently, in a ternary system, the number of ternary bits can exceed the number of gates, a crucial factor for increasing the information density. With the same number of gates, ternary logic can accommodate more information, leading to higher information density. Alternatively, to store equivalent information, ternary logic has the potential to reduce the number of required devices, pins, and connections. Ultimately, this transition can drastically decrease the overall system complexity to 63.1% [1,4,20].

The advancement of the standard ternary inverter (STI) represents a crucial foundational element in the development of ternary logic systems. Its practical implementation holds immense significance and garners substantial attention within the field. Currently, two methods exist for realizing STIs. One approach involves the direct design of the STI using MOSFETs, initially preferred due to its compatibility with the CMOS platform. However, this method typically requires a larger number of MOSFETs for an STI, ranging from three to as many as five [21,22,23]. Additionally, it necessitates multiple power supplies, including additional voltages such as –V_DD_, 1/3V_DD_, and 2/3V_DD_ [22,24], or passive elements like two additional resistors [25]. Consequently, this not only results in a larger chip area and increased power consumption, but also elevates the complexity of system design. As a result, recent years have witnessed a decline in research focused on STIs based on this approach.

An alternative approach involves constructing the STI using ternary devices that exhibit an intermediate state between the on and off states. Unlike the method mentioned earlier, this approach avoids an increase in the required device number and design complexity. However, the intermediate state, often arising from negative differential resistance (NDR) [26,27] and negative differential transconductance (NDT) [28,29], necessitates the use of heterojunctions formed by relatively novel and immature materials. Examples of such materials include BP/MoS2 [19,30], BP/ReS2/HfS2 [31], h-BN/WSe2/InSe [32], and even certain organic materials like PTCDI-C8 [33] and PTCDI-C13 [34]. Unfortunately, this reliance on novel materials renders this approach incompatible with the CMOS platform and poses challenges for mass producibility.

Obviously, based on the MOSFETs, the ternary logic inverter can be compatible with the CMOS technology, but it requires a passive component or multi-valued power supply, which makes the circuit significantly complex. The other method, with the aid of the ternary device, requires only two devices for implementing one ternary inverter and does not need multi-valued power supply, but this method relies on the novel immature materials that are incompatible with CMOS technology.

To address the issues and implement a ternary inverter compatible with the CMOS platform, a novel device combining the tunneling and drift-diffusion mechanisms is proposed. The proposed TDFETs can present three states without involving any novel immature material. Based on the TDFETs, a ternary inverter is designed and investigated. Additionally, the influences of the supply voltage as well as the key parameters of the TDFETs are studied in detail.

## 2. Device Structure and Simulation Setup

**Figure 1:**
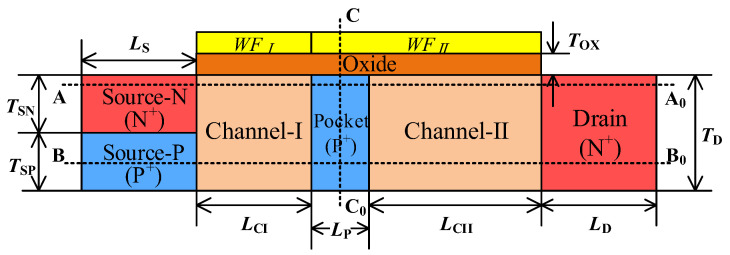
The structure of the proposed TDFET.

Figure 1 shows the device structure of the proposed TDFET. The source consists of two regions with the same concentration, but different doping types. The gate is divided into two parts, one with work function WF_I_ and the other with work function WF_II_. The corresponding channels are channel-I and channel-II. A pocket is inserted into channel-II, enabling the device to conduct a current through different mechanisms. For the convenience of subsequent analysis and description, the cut-lines that would be used later are also marked in Figure 1. The cut lines AA_0_ and BB_0_ are 15 nm and 1 nm below the oxide/channel interface. The CC_0_ is perpendicular to the channel direction and located at the midpoint of the pocket region. The device parameters adopted in the simulation are listed in Table 1.

The proposed TDFET is studied using 2-D technology computer-aided technique (TCAD) tools(Sentaurus 2013). In order to account for the arbitrary tunneling barrier with a non-uniform electrical field, the BTBT model with a dynamic nonlocal path accounting for the arbitrary tunneling barrier with a non-uniform electrical field is adopted, and the nonlocal tunneling parameters [35,36] A_path_ and B_path_ are 4 × 10^14^ cm^−3^·s^−1^ and 1.9 × 10^7^ V·cm^−1^, respectively. The Slotboom model is utilized to consider the influence of the high doping density on the band gap. Additionally, the doping-dependent mobility model, high-field velocity saturation model, and the Shockley–Read–Hall recombination models are also included.

**Figure 2:**
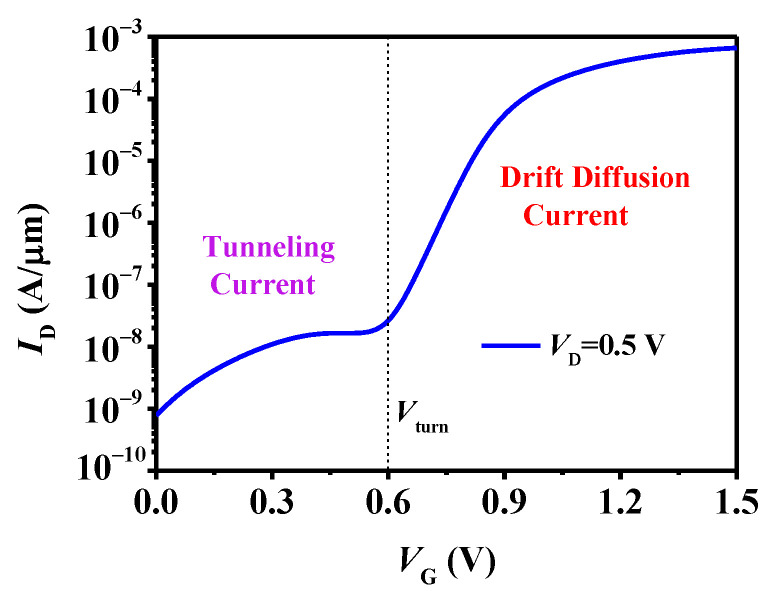
The transfer characteristics of the TDFET.

Figure 2 presents the transfer characteristics of the n-type TDFET. It is evident that the characteristics of the TDFET differ significantly from those of the conventional TFETs and MOSFETs. A noticeable transition occurs at a turn voltage (V_turn_) of about 0.6 V, resulting in three distinct states in the TDFET, namely, the off-state, the on-state, and an intermediate state between the on and off states. This tri-state behavior of the TDFET makes it highly suitable for the design of ternary logic.

**Figure 3:**
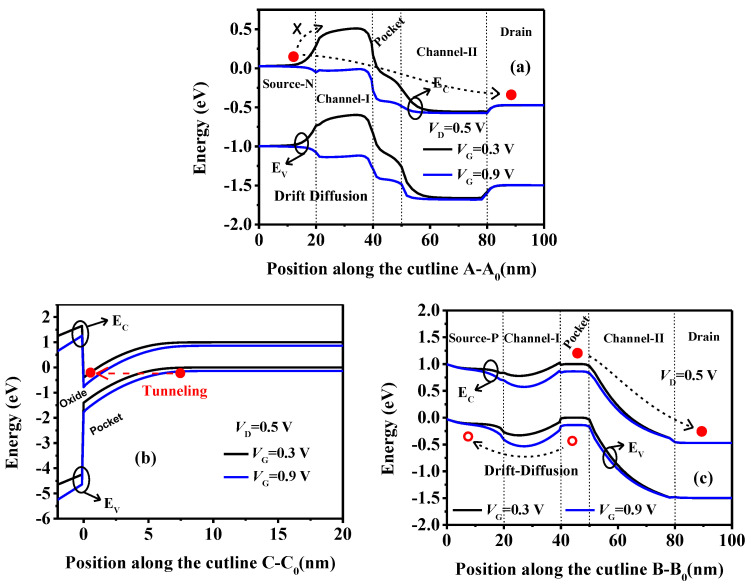
The energy band diagrams along the cutlines (**a**) AA_0_, (**b**) CC_0_, and (**c**) BB_0_ of the TDFET.

**Figure 4:**
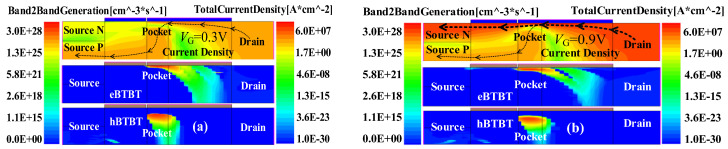
The contour mapping of the current density and BTBT rate of the TDFET device at (**a**) V_G_ = 0.3 V and (**b**) V_G_ = 0.9 V.

This tri-state behavior is actually caused by two different conduction mechanisms, as exhibited in Figure 3, showing the band diagrams along the cut-lines AA_0_, BB_0_, and CC_0_. Owing to the relatively large WF_I_, a high barrier of about 0.5 eV forms between the Source-N (SN) and channel-I regions at V_G_ = 0.3 V, as presented in Figure 3a. This barrier blocks the electrons in the SN to the drain via the drift-diffusion mechanism. However, in this case, the conduction band (E_C_) in the pocket region near the oxide overlaps with the valance band (E_V_) further away, as depicted in Figure 3b. This allows the electrons farther away from the oxide to tunnel to the region near the oxide along the cut-line CC_0_, which can be verified in Figure 4a by the high electron band-to-band tunneling rate (eBTBT) near the oxide and high hole band-to-band tunneling rate (hBTBT) a little further away from the oxide in the pocket region. The tunneling electrons near the oxide drift further right along the channel to the drain, while the holes further away from the oxide drift left to the Source-P (SP), as shown in Figure 3c, forming the current path from the drain to the source along the dotted line with the arrow in the top figure of Figure 4a.

With the increased V_G_ to 0.9 V, the E_C_ in the channel-I descends. The barrier between the SN and the channel-I regions decreases, and even to zero, as shown in Figure 3a, allowing large amount of electrons in the SN region to be thermally injected into the channel-I and further drift to the drain. It should also be pointed out that the tunneling process in the pocket region still exists as shown in Figure 3b. Therefore, in this case, the tunneling and the drift-diffusing mechanisms coexist in the device. Figure 4b gives the distribution of the current density and tunneling rate at V_G_ = 0.9 V. Obviously, there are high eBTBT and hBTBT in the pocket region. But there are many more electrons thermally injected from the SN than from the tunneling process. Thus, the drift-diffusion current is much higher than the tunneling current, and the current density is mainly distributed along the drift-diffusion current path indicated by the thicker dotted line in the top figure of Figure 4b.

Obviously, there are two conduction mechanisms in the TDFET. For V_G_ < V_turn_, the current is primarily dominated by the tunneling process in the pocket region and flows from the drain to the SP of the source. For V_G_ > V_turn_, the current is mainly governed by the drift-diffusion mechanism and flows from the drain to the SN of the source. As the drift-diffusion current is much greater than the tunneling current, a sudden increase in the current occurs near the V_turn_ and causes a noticeable transition on the transfer curve, forming the tri-state behavior of the proposed TDFET.

## 3. Ternary Inverter Based on the TDFET

**Figure 5:**
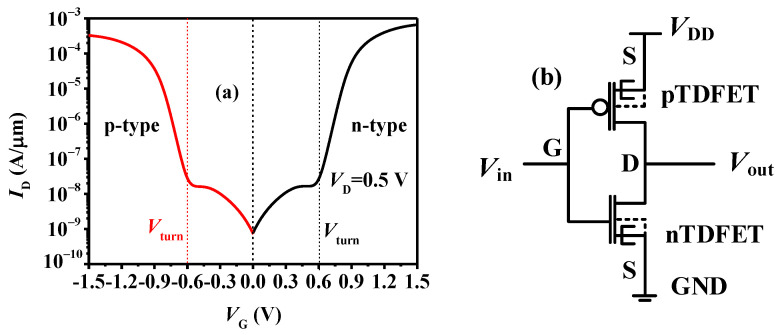
(**a**) Symmetrical characteristics of the n-type and p-type TDFETs; (**b**) circuit diagram of ternary inverter.

To implement a ternary inverter, a p-type device with symmetrical characteristics to the n-type TDFET is designed. The structure of the p-type device is identical to that of the n-type device, except for the change in the doping type in all regions. The gate work functions WF_I_ and WF_II_ of the p-type device are adjusted as 4.47 and 5.64 eV, respectively. Additionally, the pocket concentration is set as 3.7 × 10^19^ cm^−3^. The other parameters remain the same as in Table 1. Figure 5a presents the obtained symmetrical n-type and p-type curves, which are desirable features for the inverter design. Figure 5b presents the circuit diagram of the ternary inverter, showing a simple connection where the gates of the n-type and p-type devices are connected as the input (V_in_), and the drain is connected as the output (V_out_), with the source of the p-type device connected to V_DD_ (operating voltage of the inverter) and the source of the n-type device connected to GND, similarly to the binary inverter circuit.

**Figure 6:**
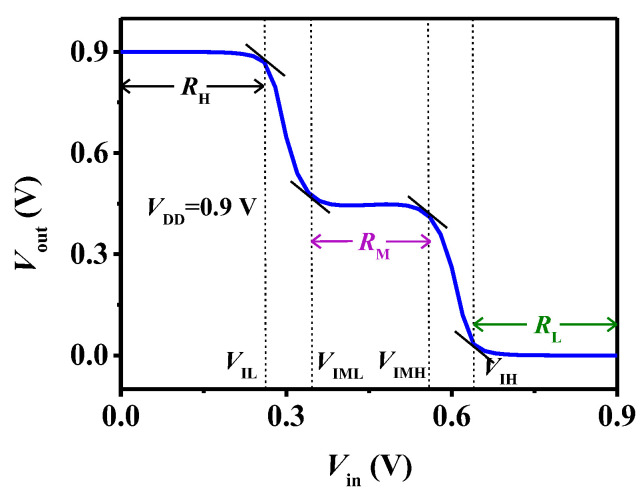
The VTC of the ternary inverter based on the TDFETs.

Figure 6 shows the voltage transfer characteristic (VTC) of the designed ternary inverter based on the TDFETs. It can be seen that the inverter exhibits three distinct output states. The V_IL_ and V_IH_ are the maximum and minimum of the input voltage and can be considered as the logic L and H, respectively. The V_IML_ and V_IMH_ are the minimum and maximum of the input voltage and can be considered as the logic M. The V_IL_, V_IH_, V_IML_, and V_IMH_ are all defined at the points where the slope equals −1. The input voltage ranges corresponding to the logic L, M, and H are calculated as R_L_ = V_IL_, R_M_ = V_IMH_ − V_IML_, and R_H_ = V_DD_ − V_IH_, respectively.

**Figure 7:**
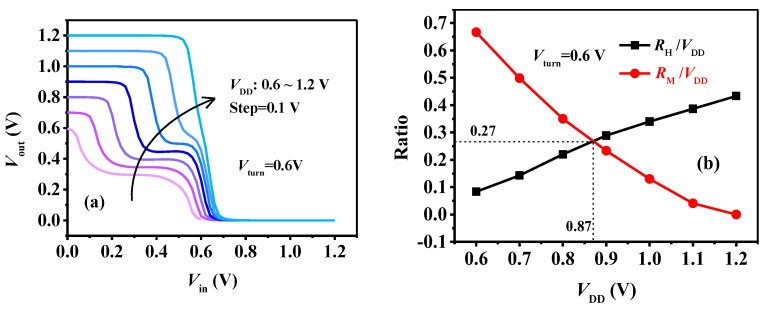
(**a**) The VTC of the ternary inverters and the (**b**) R_H_/V_DD_ and R_M_/V_DD_ varied with V_DD_.

The VTC of the ternary inverter with various V_DD_ from 0.6 V to 1.2 V is shown in Figure 7a. Interestingly, with increased V_DD_, the logic L and H show totally different changes compared to those of the intermediate logic M. The R_M_ gradually decreases, while the R_L_ and R_H_ gradually increase with V_DD_. Even when V_DD_ = 1.2 V, the intermediate logics M totally disappears and the ternary inverter becomes a conventional binary inverter. The varied R_M_ and R_H_ with V_DD_ are extracted in Figure 7b. Due to the symmetrical transfer characteristics of the p-type and n-type devices, the R_H_ ≈ R_L_ (as can also be seen in Figure 7a), and therefore, R_L_/V_DD_ is not present. We can see that as V_DD_ increases, the R_H_/V_DD_ gradually increases, while the R_M_/V_DD_ decreases. When V_DD_ = 1.45 V_turn_ = 0.87 V, R_H_ ≈ R_L_ = R_M_ = 0.27 V_DD_, and the three levels are equiprobable, which is preferred for a ternary inverter.

**Figure 8:**
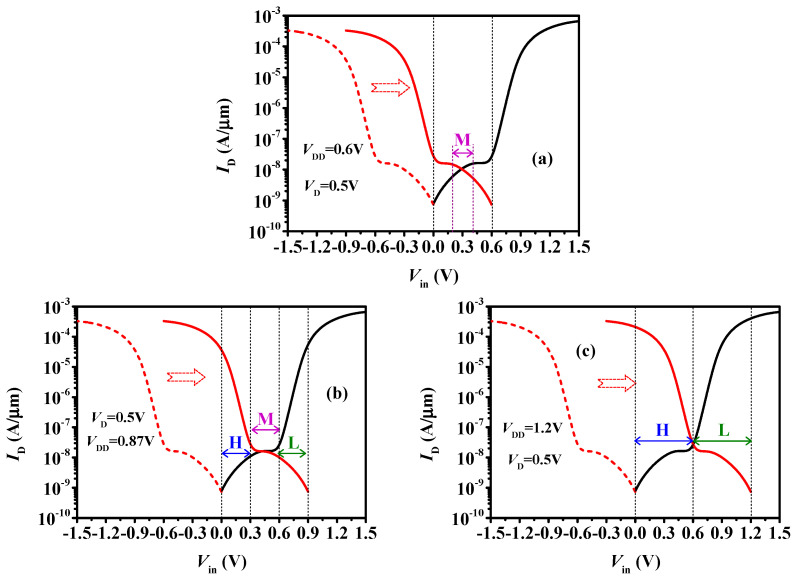
I_D_-V_in_ characteristics of the ternary inverters at V_DD_ of (**a**) 0.6 V, (**b**) 0.87 V, and (**c**) 1.2 V.

To further study the effects of V_DD_, the I_DS_ varied with the input voltage V_in_ is plotted in Figure 8. Considering that in an inverter, the gate voltage of the n-type (V_Gn_) and p-type TDFETs (V_Gp_) are V_Gn_ = V_in_ and V_Gp_ = V_in_ − V_DD_, respectively, all we need is to shift the transfer curve of the p-type TDFET by V_DD_ towards the positive direction to obtain the I_DS_-V_in_ characteristics. Figure 8a presents the I_DS_-V_in_ curves at V_DD_ = 0.6 V. It can be seen that, as the V_in_ increases from 0.0 V to 0.2 V, the p-type device exhibits a larger current than the n-type device, and the resistance of the pull-up p-type device R_up_ is smaller than the pull-down n-type device R_down_, which results in V_out_ > V_DD_/2. Although the p-type current is larger than the n-type current, the maximum difference is less than an order of magnitude. This means the R_up_ is not small enough compared with the R_down_. Therefore, the logic H cannot be built, as shown in Figure 7a. The reason why the logic L disappears can be also explained in the similar way as the increase in Vin from 0.4 V to 0.6 V. For V_in_ increasing from 0.2 V to 0.4 V, both the devices operate in the transition region between the off state and the intermediate state, where the current and resistance of the devices are comparable. Thus, the output voltage is near 0.3 V (V_DD_/2). This is why the inverter only presents the logic M at a low voltage of V_DD_ = 0.6 V_in_ (Figure 7a).

To make sure the inverter can present three distinct logics, the p-type transfer curve need to be shifted further to the right. That is to say that a larger V_DD_ is required, as shown in Figure 8b, in which V_DD_ = 0.9 V. It can be seen that when V_in_ is near 0V, the p-type TDFET is in the drift-diffusion region, while the n-type TDFET is in the off state. The R_up_ is much smaller than R_down_, and the high logic, H, forms. As for 0.3 V < V_in_ < 0.6 V, both the n-type and p-type devices operate in the tunneling current region in which the R_up_ ≈ R_down_ and the V_out_ is near the V_DD_/2. Thus, the intermediate logic, M, is obtained. When V_in_ closes to the V_DD_, the p-type device enters the off-state while the n-type device enters the drift-diffusion region. Hence, the R_up_ is much larger than the R_down_, and the V_out_ approaches 0 V, resulting in the low logic L. This explains why the inverter can present three distinct levels at a V_DD_ near 0.9 V.

However, as the V_DD_ continues to increase, the input voltage range R_M_, over which both the n-type and p-type devices are in the tunneling current region, gradually decreases. The logic M on the VTC becomes more and more narrow. Until the V_DD_ = 1.2 V, as shown in Figure 8c, the R_M_ decreases to 0 and the intermediate logic M completely disappears. While the V_in_ rises from 0.0 V to V_DD_, there are only two distinct logics. Namely, when 0.0 V < V_in_ < V_DD_/2, the p-type current is far larger than the n-type current and the R_up_ < R_down_ leading to logic H. When V_DD_/2 < V_in_ < V_DD_, the p-type current is far smaller than the n-type current and the R_up_ > R_down_, resulting in logic L. Obviously, in this case, the ternary inverter becomes a conventional binary inverter.

Based on the analysis presented in Figure 8, it is evident that V_DD_ significantly influences the performance of the inverter. If we aim to design a ternary inverter with R_L_=R_M_=R_H_, we can deduce from Figure 8b that V_turn_ = (2/3)V_DD_, considering R_L_ + R_M_ + R_H_ = V_DD_. In other words, the theoretical V_DD_ for an ideal equiprobable ternary inverter is V_DD_ = 1.5 V_turn_. However, the practical scenario is that R_L_ + R_M_ + R_H_ < V_DD_ due to transitions between different logic states. This discrepancy is the reason why the equiprobable ratio R_H_/V_DD_ = R_M_/V_DD_ = 0.27 in Figure 7b is smaller than the ideal value of 1/3. Additionally, it explains why the V_DD_ required for the equiprobable case is 0.87 V, slightly less than the theoretical value of 1.5 Vturn (0.9 V).

**Figure 9:**
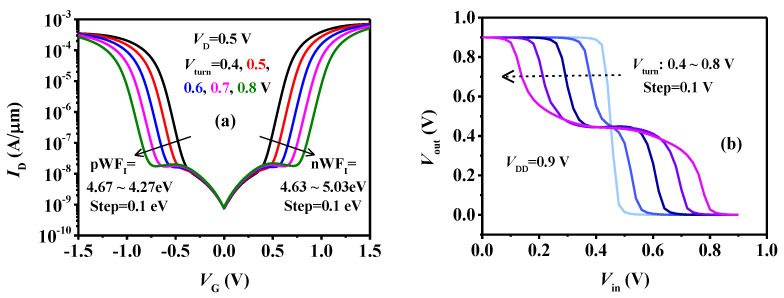
(**a**) Transfer characteristics of the TDFETs and (**b**) the VTCs for different WF_I_.

**Figure 10:**
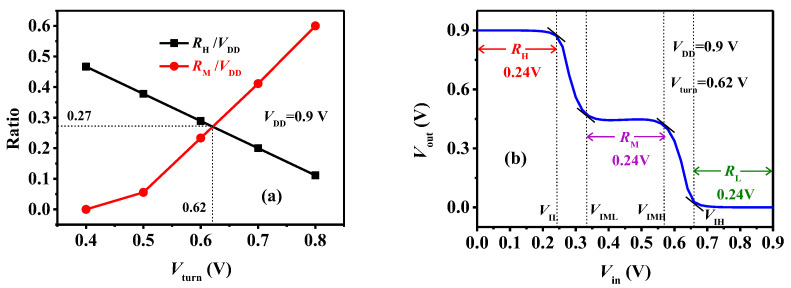
(**a**) The R_H_/V_DD_ and R_M_/V_DD_ varied with V_turn_ and (**b**) the equiprobable VTC at V_DD_ = 0.9 V.

Obviously, we have the flexibility to adjust V_turn_ to achieve an equiprobable inverter for a specified V_DD_ of 0.9 V. Considering that the V_turn_ is the critical gate voltage over which the drift-diffusion mechanism starts to dominate the device current, we can modulate the WF_I_ to change the V_turn_. Figure 9a depicts the symmetrical transfer curves of the TDFETs with different WF_I_. We can see that the absolute value of V_turn_ increases with the decreased WF_I_ for the p-type TDFET and the increased WF_I_ for the n-type device. As the absolute value of V_turn_ increases, the intermediate state of the transfer curve becomes flatter and more obvious. Thus, the input voltage range R_M_ over which both the devices are in the intermediate state increases and the logic M gradually widens, as presented in Figure 9b, giving the variations in the VTC with V_turn_ at fixed V_DD_ = 0.9 V. Figure 10a exhibits the varied R_H_/V_DD_ and R_M_/V_DD_ with V_turn_, and we can see that, for V_DD_ = 0.9 V, the ternary inverter becomes equiprobable at V_turn_ = 0.62 V. In this case, R_H_ = R_M_ ≈ 0.27 V_DD_. The corresponding VTC is plotted in Figure 10b. R_M_ = R_H_ = R_L_ = 0.24 V indicates that the ternary inverter is equiprobable, and the subsequent simulations are based on this inverter.

**Figure 11:**
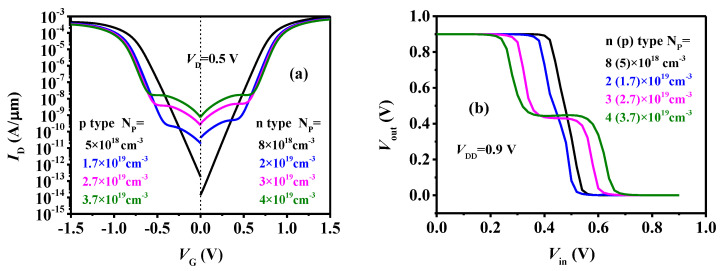
(**a**) The transfer curves of the TDFETs and (**b**) VTC of the ternary inverter with different N_P_.

In a TDFET, the tunneling process happens in the pocket region. Thus, the pocket doping density N_P_ and pocket length L_P_ may show important effects. The symmetrical transfer curves of the n-type and p-type TDFETs with different N_P_ are given in Figure 11a. It can be observed that, as the N_P_ decreases, the tunneling-dominated current decreases, while the drift-diffusion-dominated current is hardly affected. This results in decreased V_turn_ and a weakened intermediate state between the on and off states, which further leads to gradually narrowing logic M on the VTC curve, as depicted in Figure 11b, which shows the varied VTC curves with N_P_. Until the N_P_ decreases to 8 × 10^18^ cm^−3^ for the n-type device and 5 × 10^18^ cm^−3^ for the p-type device, the tunneling current, as well as the tri-state behavior, completely disappear, and the characteristics of the TDFET become almost identical to those of MOSFETs. In this case, the logic M on the VTC curve also completely disappears, and the ternary inverter becomes a conventional binary inverter.

**Figure 12:**
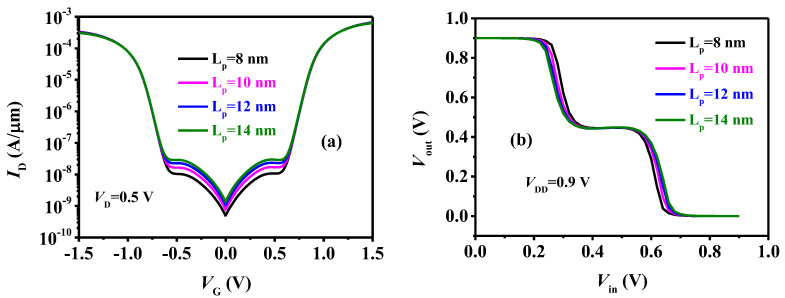
(**a**) The transfer curves of the TDFETs and (**b**) VTC of the ternary inverter with different L_P_.

The influence of L_P_ is illustrated in Figure 12. As L_P_ increases, the effective tunneling area and the tunneling current gradually increase. However, the drift-diffusion current, primarily influenced by the barrier between the SN and channel-I regions, exhibits almost no variation. Consequently, this results in a slight increase in V_turn_, as depicted in Figure 10a, and a subsequent slight widening of the logic M on the VTC, as shown in Figure 10b.

**Figure 13:**
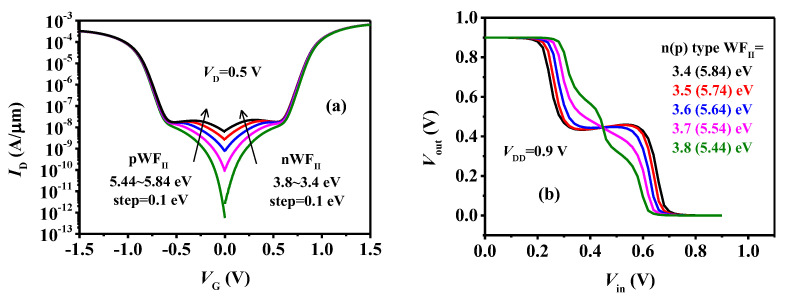
(**a**) The transfer curves of the TDFETs and (**b**) VTC of the ternary inverter with different WF_II_.

Figure 13 illustrates the impact of WF_II_. As the WF_II_ increases for the p-type device and decreases for the n-type device, the tunneling current undergoes a gradual increase, resulting in a flatter intermediate state on the transfer curve, depicted in Figure 13a. Referencing the analysis in Figure 8, it becomes apparent that the flatter intermediate state contributes to a wider and flatter logic M, as shown in Figure 13b.

**Figure 14:**
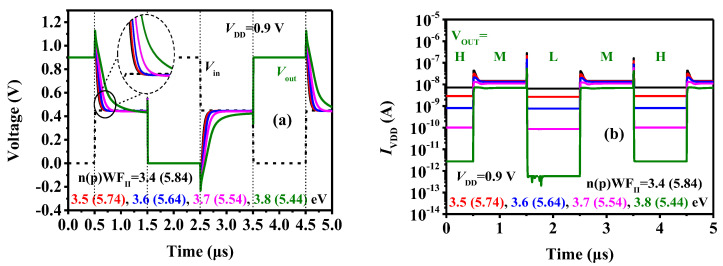
(**a**) Inverter’s transient characteristics and (**b**) current curves with different WF_II_.

Figure 14a illustrates how WF_II_ impacts the transient characteristics of the inverter. With changes in WF_II_, the tunneling current gradually decreases, leading to longer charging and discharging times for the capacitance. Consequently, the delay time for the inverter output to transition from level H to level M increases from 0.077 μs to 0.321 μs, causing a decrease in the inverter speed. Moreover, the variation in the intermediate state current affects the circuit power consumption. Figure 14b depicts the current variation from the power supply to the ground during the inverter output switching between different levels. It is evident that when the inverter outputs levels H and L, the power supply current is relatively small, whereas for level M, the power supply current is larger. This is attributed to the fact that at levels H and L, one of the n-type and p-type devices conducts while the other is turned off, resulting in no direct current path from the power supply to the ground. However, at level M, both n-type and p-type devices are in a partially conducting state, leading to the inverter power consumption being the sum of the n-type and p-type power consumption. Furthermore, as nWF_II_ decreases and pWF_II_ increases, both the intermediate state current and the off-state current of the device increase, resulting in a gradual increase in the inverter current. In conclusion, WF_II_ significantly impacts the stability, speed, and power consumption of the inverter’s intermediate state. In practical design, inverters must balance stability, speed, and power consumption based on the circuit’s application scenarios.

## 4. Conclusions

In this paper, a novel TDEFT is proposed based on the band-to-band tunneling and drift-diffusion processes. Owing to the hybrid conduction mechanism, the TDFET shows tri-state behavior and is adopted to build a ternary inverter, which presents three distinct levels. The implemented ternary inverter, without involving any novel immature material, requires only two TDFET devices and is compatible with the CMOS platform. The operation principle of the ternary inverter is investigated, and the result is that that the V_DD_ shows significant influence on the inverter performance. When the operating voltage V_DD_ = 1.45 V_turn_, the three states of the ternary inverter have approximately equal probability. Furthermore, the effects of the key device parameters, such as the pocket doping density, pocket length, and gate work function, are also discussed.

## Figures and Tables

**Table 1:** The device parameters used in the simulation.

Parameters	Symbols	Values
Source N Thickness	*T*_SN_	10 nm
Source P Thickness	*T*_SP_	10 nm
Source N Doping	*N*_SN_	1 × 10^19^ cm^−3^
Source P Doping	*N*_SP_	1 × 10^19^ cm^−3^
Channel I Length	*L*_CI_	20 nm
Channel I Doping	*N*_CI_	1 × 10^16^ cm^−3^
Channel II Length	*L*_CII_	30 nm
Channel II Doping	*N*_CII_	1 × 10^16^ cm^−3^
Pocket Length	*L*_P_	10 nm
Pocket Doping	*N*_P_	4 × 10^19^ cm^−3^
Drain Doping	*N*_D_	1 × 10^19^ cm^−3^
Gate Oxide Thickness	*T*_OX_	2 nm
Oxide Dielectric Constant	*ε*	22
Gate Work Function I	*WF*_I_	4.83 eV
Gate Work Function II	*WF*_II_	3.60 eV

## Data Availability

The original contributions presented in the study are included in the article, further inquiries can be directed to the corresponding author.
